# Supplementary material for: MIGOU: A Low-Power Experimental Platform with Programmable Logic Resources and Software-Defined Radio Capabilities
Source: Sensors (Basel). 2019 Nov 15;19(22):4983. doi: 10.3390/s19224983 (PMC6891619; doi:10.3390/s19224983)
Supplement: Supplementary file 1 [file sensors-19-04983-s001.zip › MIGOU-Bill of Materials.pdf]

Bill of Materials

Migou Platform

Source Data From: **MIGOU.PrjPcb**
Project: **MIGOU.PrjPcb**
Variant: **None**

Creation Date: 12/06/2017 19:02:37
Print Date: 12-Jun-17 7:05:09 PM

Designator	Quan	Value	Footprint	Manufacturer 1	Manufacturer Part Number	Supplier 1	Supplier Part Number 1	Description 1
BL1	1		2450FB15A050E	Johanson Technology Inc.	2450FB15A050E	Digi-Key	712-1065-1-ND	FILTER BALUN CER CHIP 2.4GHZ
BTN-1, BTN-2	2		Push button SMD 2 leads	C&K	RS-282G05A3-SM RT	Digi-Key	CKN10384CT-ND	SWITCH TACTILE SPST-NO 0.05A 12V
C1, C2, C3, C4, C5, C6, C7, C8, C9, C10, C11, C12, C13, C40, C41, C45, C47, C48, C53, C54, C57, C58, C62, C63, C68, C69, C74, C101, C132, C149, C154, C173, C189, C191, C192, C196, C197, C198	38	10n	0402 CAP	Murata Electronics North America	GRM155R71C103KA01D	Digi-Key	490-1313-1-ND	CAP CER 10000PF 16V X7R 0402
C14, C15, C16, C17, C18, C19, C20, C21, C22, C23, C24, C25, C26, C42, C43, C49, C50, C51, C55, C59, C60, C64, C65, C67, C72, C73, C75, C76, C79, C83, C84, C85, C86, C87, C102, C104, C105, C106, C107, C108, C109, C111, C113, C115, C137, C141, C145, C176, C178, C180, C182, C184, C186, C188, C193, C194, C199, C200, C201	59	100n	0402 CAP	Murata Electronics North America	GRM155R71C104KA88D	Digi-Key	490-3261-1-ND	CAP CER 0.1UF 16V X7R 0402
C27, C28, C29, C30, C31, C32, C33, C34	8	100p	0402 CAP	Wurth Electronics Inc.	885012205019	Digi-Key	732-7514-1-ND	CAP CER 100PF 16V X7R 0402
C35	1	100u	CAP POL SMD 6.6x6.6	Wurth Electronics Inc.	875105344010	Digi-Key	732-6425-1-ND	CAP ALUM POLY 100UF 20% 16V SMD
C36, C37, C38	3	220u	CAP POL SMD 6.6x6.6	Wurth Electronics Inc.	875105244013	Digi-Key	732-6413-1-ND	CAP ALUM POLY 220UF 20% 10V SMD
C39	1	330u	CAP POL SMD 6.6x6.6	Wurth Electronics Inc.	875105245015	Digi-Key	732-6415-1-ND	CAP ALUM POLY 330UF 20% 10V SMD
C44, C46, C52, C56, C61, C66, C190, C195	8	10u	0603 Cap	Murata Electronics North America	GRM188R60J106ME47D	Digi-Key	490-3896-1-ND	CAP CER 10UF 6.3V X5R 0603
C70, C71	2	47p	0402 CAP	Yageo	CC0402JRNPO9BN470	Digi-Key	311-1021-1-ND	CAP CER 47PF 50V NPO 0402
C77, C139, C143, C147, C150, C174, C175, C177, C179, C181, C183, C185, C187	13	22u	0805 CAP	Samsung Electro-Mechanics Ame	CL21A226KPCLRNC	Digi-Key	1276-6786-1-ND	CAP CER 22UF 10V X5R 0805
C78, C98, C99	3	2.2p	0402 CAP	Murata Electronics North America	GRM1555C1H2R2BA01D	Digi-Key	490-6227-1-ND	CAP CER 2.2PF 50V NP0 0402
C80	1	1n	0402 CAP	KEMET	C0402C102K8RACTU	Digi-Key	399-7757-1-ND	CAP CER 1000PF 10V X7R 0402
C81, C116, C117	3	10p	0402 CAP	AVX Corporation	04025U100CAT2A	Digi-Key	478-5991-1-ND	CAP CER 10PF 50V NP0 0402
C82, C120, C125, C136, C140, C144, C148, C172	8	10u	0805 CAP	Murata Electronics North America	GRM21BR71A106KA73L	Digi-Key	490-10516-1-ND	CAP CER 10UF 10V X7R 0805
C88, C89, C90	3	1u	0402 CAP	KEMET	C0402C105K9PACTU	Digi-Key	399-4873-1-ND	CAP CER 1UF 6.3V X5R 0402

C91, C92	2	1.8p	0402 CAP	Murata Electronics North America	GRM1555C1H1R8WA01D	Digi-Key	490-6216-1-ND	CAP CER 1.8PF 50V NP0 0402
C93	1	0.8p	0402 CAP	Murata Electronics North America	GRM1555C1HR80WA01D	Digi-Key	490-6270-1-ND	CAP CER 0.8PF 50V NP0 0402
C94, C95	2	1.2p	0402 CAP	Murata Electronics North America	GRM1555C1H1R2WA01D	Digi-Key	490-6210-1-ND	CAP CER 1.2PF 50V NP0 0402
C96	1	15p	0402 CAP	Murata Electronics North America	GRM1555C1H150FA01D	Digi-Key	490-6200-1-ND	CAP CER 15PF 50V NP0 0402
C97	1	8.2p	0402 CAP	Murata Electronics North America	GRM1555C1H8R2WA01D	Digi-Key	490-10689-1-ND	CAP CER 8.2PF 50V NP0 0402
C100	1	4.7n	0402 CAP	Murata Electronics North America	GRM155R71C472KA01D	Digi-Key	490-6334-1-ND	CAP CER 4700PF 16V X7R 0402
C103, C110, C112, C114, C162	5	4.7u	0603 Cap	Samsung Electro-Mechanics Ame	CL10B475KQ8NQNC	Digi-Key	1276-2087-1-ND	CAP CER 4.7UF 6.3V X7R 0603
C118	1	10u	0805 CAP	Samsung Electro-Mechanics Ame	CL21B106KOQNNNE	Digi-Key	1276-2872-1-ND	CAP CER 10UF 16V X7R 0805
C119, C121, C128	3	4.7u	0805 CAP	Samsung Electro-Mechanics Ame	CL21B475KPFNNNE	Digi-Key	1276-2972-1-ND	CAP CER 4.7UF 10V X7R 0805
C122	1	10n	0603 Cap	Murata Electronics North America	GRM188R71H103JA01D	Digi-Key	490-9734-1-ND	CAP CER 10000PF 50V X7R 0603
C123, C126	2	47u	0805 CAP	Samsung Electro-Mechanics Ame	CL21A476MQCLRNC	Digi-Key	1276-2420-1-ND	CAP CER 47UF 6.3V X5R 0805
C124, C164, C167	3	1u	0603 Cap	Samsung Electro-Mechanics Ame	CL10B105KP8NNNC	Digi-Key	1276-1946-1-ND	CAP CER 1UF 10V X7R 0603
C127	1	0.47u	0603 Cap	Samsung Electro-Mechanics Ame	CL10B474KO8NNNC	Digi-Key	1276-1062-1-ND	CAP CER 0.47UF 16V X7R 0603
C129, C130, C131, C135, C153, C155, C156, C157, C158, C159, C160, C161, C163, C168, C169, C170, C171, C207, C209	19	100n	0603 Cap	Samsung Electro-Mechanics Ame	CL10B104KB8SFNC	Digi-Key	1276-1936-1-ND	CAP CER 0.1UF 50V X7R 0603
C133, C151	2	150u	1206 CAP	Murata Electronics North America	GRM31CR60J157ME11L	Digi-Key	490-13969-1-ND	CAP CER 150UF 6.3V X5R 1206
C134, C152	2	1000u	CAP POL 10x12.5 Pitch 5mm	Nichicon	UVR1A102MPD	Digi-Key	493-1025-ND	CAP ALUM 1000UF 20% 10V RADIAL
C138, C142, C146	3	3.3n	0402 CAP	Murata Electronics North America	GRM155R71H332JA01D	Digi-Key	490-10780-1-ND	CAP CER 3300PF 50V X7R 0402
C165, C166, C203, C204	4	20p	0402 CAP	Murata Electronics North America	GRM1555C1H200GA01D	Digi-Key	490-6217-1-ND	CAP CER 20PF 50V NP0 0402
C202	1	47u	CAP POL SMD 6.6x6.6	Wurth Electronics Inc.	875105344006	Digi-Key	732-6421-1-ND	CAP ALUM POLY 47UF 20% 16V SMD
C205, C206	2		0402 CAP					
C208	1	100p	0603 Cap	Samsung Electro-Mechanics Ame	CL10B101KB8NNNC	Digi-Key	1276-1909-1-ND	CAP CER 100PF 50V X7R 0603
CMC1	1		DLW21SN900SQ2L	Murata Electronics North America	DLW21SN900SQ2L	Digi-Key	490-5137-1-ND	CMC 330MA 2LN 90 OHM SMD
CN-1	1		Header-1x3-Jumper					
CN-2	1		HEADER-2X3					
CN-3	1		HEADER-2X5 JTAG	On Shore Technology Inc.	302-S101	Digi-Key	ED1543-ND	CONN HEADER VERT 10POS GOLD
CN-4	1		Header-1x3					
CN-5, CN-6	2		Bat-Con-S2B-PH-K-S	JST Sales America Inc.	S2B-PH-K-S(LF)(SN)	Digi-Key	455-1719-ND	CONN HEADER PH SIDE 2POS 2MM
CN-7, CN-8	2		HEADER-1x2-Jumper					
CN-9, CN-10	2		Header-2x12					
CV1	1			Wurth Electronics Inc.	36003250	Digi-Key	732-2491-ND	
DC-1	1		DC-Conn-RAPC 722	Switchcraft Inc.	RAPC722X	Digi-Key	SC1313-ND	CONN PWR JACK 2X5.5MM SOLDER
F1, F2, F3	3		FIDUCIAL					
FB1	1	120 Ohm @ 100 MHz	0603 Ferrite	Murata Electronics North America	BLM18PG121SN1D	Digi-Key	490-1037-1-ND	FERRITE BEAD 120 OHM 0603 1LN
FB2	1	220 Ohm @ 100 MHz	0603 Ferrite	Wurth Electronics Inc.	74279263	Digi-Key	732-1581-1-ND	FERRITE BEAD 220 OHM 0603 1LN
FB3, FB4, FB5	3	600 Ohm @ 100 MHz	0603 Ferrite	Murata Electronics North America	BLM18KG601SN1D	Digi-Key	490-5258-1-ND	FERRITE BEAD 600 OHM 0603 1LN
FR1	1		WE36103255	Wurth Electronics Inc.	36103255	Digi-Key	732-2489-ND	
L1	1	5.6n	0402 IND	Johanson Technology Inc.	L-07C5N6SV6T	Digi-Key	712-1462-1-ND	FIXED IND 5.6NH 300MA 270 MOHM
L2	1	2.2u	IND-2.2UH-IFSC1515AHER2R2M01	Vishay Dale	IFSC1515AHER2R2M01	Digi-Key	541-1404-1-ND	FIXED IND 2.2UH 3A 45 MOHM SMD
L3, L8	2	1u	0806 Ind	Bourns Inc.	SRP2010-1R0M	Digi-Key	SRP2010-1R0MCT-ND	FIXED IND 1UH 2.1A 72 MOHM SMD
L4, L5, L6, L7, L15	5	2.2u	IND-2.2UH-4020	Wurth Electronics Inc.	74438356022	Digi-Key	732-8232-1-ND	FIXED IND 2.2UH 4.7A 35 MOHM SMD
L9, L10, L11, L12, L13, L14, L16	7	100n	0603 IND	Panasonic Electronic Components	ELJ-RER10JFA	Digi-Key	PCD1985CT-ND	FIXED IND 100NH 200MA 1.8 OHM
LD1, LD3	2		0603 LED-Green	Kingbright	APT1608ZGC	Digi-Key	754-1126-1-ND	LED GREEN CLEAR 0603 SMD
LD2, LD6	2		0603 LED-BLUE	Kingbright	APT1608QBC/D	Digi-Key	754-1434-1-ND	LED BLUE CLEAR 0603 SMD
LD4	1		0603 LED-RED	Kingbright	APT1608SECK	Digi-Key	754-1120-1-ND	LED ORANGE CLEAR 0603 SMD

LD5	1		0603 LED-RED	Kingbright	APT1608EC	Digi-Key	754-1117-1-ND	LED RED CLEAR 0603 SMD
MicroSD-1	1		MicroSD	Hirose Electric Co Ltd	DM3AT-SF-PEJM5	Digi-Key	HR1964CT-ND	CONN MICRO SD CARD PUSH-PUSH R/A
P1	1		U.FL-R-SMT-1	Hirose Electric Co Ltd	U.FL-R-SMT-1(10)	Digi-Key	H11891CT-ND	CONN UMC JACK STR 50 OHM SMD
Q1	1		SOT-23-3	Diodes Incorporated	DMP3056L-7	Digi-Key	DMP3056L-7DICT-ND	MOSFET P-CH 30V 4.3A SOT23
Q2, Q3	2		PowerPAK SO-8	Vishay Siliconix	SI7997DP-T1-GE3	Digi-Key	SI7997DP-T1-GE3CT-ND	MOSFET 2P-CH 30V 60A PPAK SO-8
R1, R12, R15, R17, R25, R26, R27, R91	8	10k	0402 RES	Panasonic Electronic Components	ERJ-2RKF1002X	Digi-Key	P10.0KLCT-ND	RES SMD 10K OHM 1% 1/10W 0402
R2, R19	2	100	0402 RES	Vishay Dale	CRCW0402100RFKEDHP	Digi-Key	541-100YCT-ND	RES SMD 100 OHM 1% 1/5W 0402
R3, R4, R5, R6	4	1k	0402 RES	Panasonic Electronic Components	ERJ-2RKF1001X	Digi-Key	P1.00KLCT-ND	RES SMD 1K OHM 1% 1/10W 0402
R7, R8, R9, R10	4	33	0402 RES	Panasonic Electronic Components	ERJ-2RKF33R0X	Digi-Key	P33.0LCT-ND	RES SMD 33 OHM 1% 1/10W 0402
R11	1	220	0402 RES	Samsung Electro-Mechanics Ame	RC1005F221CS	Digi-Key	1276-3963-1-ND	RES SMD 220 OHM 1% 1/16W 0402
R13	1	100k	0402 RES	Panasonic Electronic Components	ERJ-2RKF1003X	Digi-Key	P100KLCT-ND	RES SMD 100K OHM 1% 1/10W 0402
R14, R16, R31, R32	4	0	0402 RES	Panasonic Electronic Components	ERJ-2GE0R00X	Digi-Key	P0.0JCT-ND	RES SMD 0.0OHM JUMPER 1/10W 0402
R18	1	390k	0402 RES	Panasonic Electronic Components	ERJ-2RKF3903X	Digi-Key	P390KLCT-ND	RES SMD 390K OHM 1% 1/10W 0402
R20, R68, R69, R82	4	0	0603 RES	Panasonic Electronic Components	ERJ-3GEY0R00V	Digi-Key	P0.0GCT-ND	RES SMD 0.0OHM JUMPER 1/10W 0603
R21, R22, R74, R81	4	330	0603 Res	Yageo	RC0603FR-07330RL	Digi-Key	311-330HRCT-ND	RES SMD 330 OHM 1% 1/10W 0603
R23	1	2.2k	0402 RES	Panasonic Electronic Components	ERJ-2RKF2201X	Digi-Key	P2.20KLCT-ND	RES SMD 2.2K OHM 1% 1/10W 0402
R24	1	27	0402 RES	Panasonic Electronic Components	ERJ-2RKF27R0X	Digi-Key	P27.0LCT-ND	RES SMD 27 OHM 1% 1/10W 0402
R28	1	12k	0402 RES	Panasonic Electronic Components	ERJ-2RKF1202X	Digi-Key	P12.0KLCT-ND	RES SMD 12K OHM 1% 1/10W 0402
R29	1	39k	0603 Res	Panasonic Electronic Components	ERJ-3EKF3902V	Digi-Key	P39.0KHCT-ND	RES SMD 39K OHM 1% 1/10W 0603
R30, R53	2		0603 Res					
R33	1	28k	0603 Res	Panasonic Electronic Components	ERJ-3EKF2802V	Digi-Key	P28.0KHCT-ND	RES SMD 28K OHM 1% 1/10W 0603
R34, R41, R44, R54, R55, R70, R71, R72	8	10k	0603 Res	Panasonic Electronic Components	ERJ-3EKF1002V	Digi-Key	P10.0KHCT-ND	RES SMD 10K OHM 1% 1/10W 0603
R35	1	110	0402 RES	Panasonic Electronic Components	ERJ-2RKF1100X	Digi-Key	P110LCT-ND	RES SMD 110 OHM 1% 1/10W 0402
R36, R37	2	1.1k	0603 Res	Panasonic Electronic Components	ERJ-3EKF1101V	Digi-Key	P1.10KHCT-ND	RES SMD 1.1K OHM 1% 1/10W 0603
R38	1	4.53k	0603 Res	Samsung Electro-Mechanics Ame	RC1608F4531CS	Digi-Key	1276-4708-1-ND	RES SMD 4.53K OHM 1% 1/10W 0603
R39	1	8.45k	0603 Res	Samsung Electro-Mechanics Ame	RC1608F8451CS	Digi-Key	1276-4738-1-ND	RES SMD 8.45K OHM 1% 1/10W 0603
R40	1	330	0402 RES	Panasonic Electronic Components	ERJ-2RKF3300X	Digi-Key	P330LCT-ND	RES SMD 330 OHM 1% 1/10W 0402
R42, R43, R48, R49, R50, R51, R56, R59, R62, R65, R75, R76, R77, R78, R79, R80, R83, R84, R85, R88, R89, R90	22	100k	0603 Res	Yageo	RC0603FR-07100KL	Digi-Key	311-100KHRCT-ND	RES SMD 100K OHM 1% 1/10W 0603
R45, R46	2	100	0603 Res	Panasonic Electronic Components	ERJ-3EKF1000V	Digi-Key	P100HCT-ND	RES SMD 100 OHM 1% 1/10W 0603
R47	1	0.01	0603 Res	Panasonic Electronic Components	ERJ-3LWFR010V	Digi-Key	P19180CT-ND	RES SMD 0.01 OHM 1% 1/4W 0603
R52	1	0.2	0603 Res	Panasonic Electronic Components	ERJ-3BSFR20V	Digi-Key	P17453CT-ND	RES SMD 0.2 OHM 1% 1/4W 0603
R57, R60	2	470k	0603 Res	Panasonic Electronic Components	ERJ-3EKF4703V	Digi-Key	P470KHCT-ND	RES SMD 470K OHM 1% 1/10W 0603
R58, R61	2	150k	0603 Res	Panasonic Electronic Components	ERJ-3EKF1503V	Digi-Key	P150KHCT-ND	RES SMD 150K OHM 1% 1/10W 0603
R63	1	510k	0603 Res	Panasonic Electronic Components	ERJ-3EKF5103V	Digi-Key	P510KHCT-ND	RES SMD 510K OHM 1% 1/10W 0603
R64	1	240k	0603 Res	Panasonic Electronic Components	ERJ-3EKF2403V	Digi-Key	P240KHCT-ND	RES SMD 240K OHM 1% 1/10W 0603
R66	1	60.4	0603 Res	Panasonic Electronic Components	ERJ-3EKF60R4V	Digi-Key	P60.4HCT-ND	RES SMD 60.4 OHM 1% 1/10W 0603
R67	1	0.62	0603 Res	Panasonic Electronic Components	ERJ-3BQFR62V	Digi-Key	P17403CT-ND	RES SMD 0.62 OHM 1% 1/4W 0603
R86	1	10.2k	0603 Res	Vishay Dale	CRCW060310K2FKEA	Digi-Key	541-10.2KHCT-ND	RES SMD 10.2K OHM 1% 1/10W 0603
R87	1	20.5k	0603 Res	Vishay Dale	CRCW060320K5FKEA	Digi-Key	541-20.5KHCT-ND	RES SMD 20.5K OHM 1% 1/10W 0603
R92	1		0402 RES					
R93, R94, R95, R96, R97, R98, R99	7	1	0402 RES	Panasonic Electronic Components	ERJ-2GEJ1R0X	Digi-Key	P1.0JCT-ND	RES SMD 1 OHM 5% 1/10W 0402
R100	1	0	0805 Res	Panasonic Electronic Components	ERJ-6GEY0R00V	Digi-Key	P0.0ACT-ND	RES SMD 0.0 OHM JUMPER 1/8W 0805
SMA-1, SMA-2	2		Jack SMA 142-0701-801	Cinch Connectivity Solutions John	142-0701-801	Digi-Key	J502-ND	CONN SMA JACK 50 OHM EDGE MNT
SW-1	1		WE01014042	Wurth Electronics Inc.	450301014042	Digi-Key	450301014042-ND	SWITCH SLIDE SPDT 500MA 12V
SW-2	1		219-3MSTR	CTS Electrocomponents	219-3MSTR	Digi-Key	CT3094CT-ND	SWITCH SLIDE DIP SPST 100MA 20V

SW-3	1		WE02014072	Wurth Electronics Inc.	450302014072	Digi-Key	450302014072-ND	SWITCH SLIDE SPDT 500MA 12V
TCXO1	1	26.0 MHz	ASTXR-12-26.000MHz-511741	Abracon LLC	ASTXR-12-26.000MHZ-511741	Digi-Key	535-12689-ND	OSC TCXO 26.000MHZ CLIP SNWV SMD
TP1, TP2, TP6, TP7, TP8, TP9, TP10, TP11, TP12, TP13, TP14, TP15, TP16, TP17, TP19, TP20	16		Test Point 0.9mm					
TP3, TP4, TP5, TP21, TP22, TP23, TP24	7		Test Point 0.3mm					
U1	1		MCSM-FG-484	Microsemi Corporation	M2S050-FGG484	Digi-Key	1100-1224-ND	IC FPGA SOC 50K LUTS 484FBGA
U2	1		SOT-23-3	Richtek USA Inc.	RT9818C-29GV	Digi-Key	1028-1020-1-ND	IC VOLT DETECTOR 2.9V SOT23-3
U3	1		QFN-32	FTDI, Future Technology Devices	FT232RQ-REEL	Digi-Key	768-1008-1-ND	IC USB FS SERIAL UART 32-QFN
U4	1		QFN-48	Microchip Technology	AT86RF215-ZUR	Digi-Key	AT86RF215-ZURCT-ND	IC RF TXRX 802.15.4/ISM 48-VFQFN
U5	1		FT232HQ	FTDI, Future Technology Devices	FT232HQ-REEL	Digi-Key	768-1102-1-ND	IC HS USB TO UART/FIFO 48QFN
U6	1		93LC56BT-I/OT	Microchip Technology	93LC56BT-I/OT	Digi-Key	93LC56BT-I/OTCT-ND	IC EEPROM 2KBIT 2MHZ SOT23-6
U7	1		QFN-24	Texas Instruments	BQ24166RGET	Digi-Key	296-36560-1-ND	IC BATT CHRG MGMT LI-ION 24VQFN
U8	1		DRZ12-1950X2450TP	Texas Instruments	BQ27441DRZR-G1A	Digi-Key	296-39941-1-ND	IC BATT FUEL GAUGE LI-ION 12SON
U9, U10	2		SOT-23-5	Maxim Integrated	MAX9938FEUK+T	Digi-Key	MAX9938FEUK+TCT-ND	IC OPAMP CURR SENS 60KHZ SOT23-5
U11, U12, U13	3		QFN-16	Texas Instruments	TPS62150AQRGTTQ1	Digi-Key	296-37879-1-ND	IC REG BUCK ADJ 1A SYNC 16QFN
U14	1		TPS6209718	Texas Instruments	TPS6209718RWKT	Digi-Key	296-43331-1-ND	IC REG BUCK 1.8V 2A 11VQFN
U15	1		USBLC6-2SC6	STMicroelectronics	USBLC6-2SC6	Digi-Key	497-5235-1-ND	TVS DIODE 5.25VWM 17VC SOT23-6
U16	1		LQFP-64	STMICROELECTRONICS	STM32L496RGT6	Farnell	2725138	STMICROELECTRONICS - STM32L496RGT6 - MCU, ARM CORTEX-M4, 80MHZ, LQFP-64
U17	1		SOT323-5L	STMicroelectronics	USBDF01W5	Digi-Key	497-3086-1-ND	FILTER RC(PI) 33 OHM/47PF SMD
U18	1		MSOP-8	Microchip Technology	AT30TS75A-XM8M-T	Digi-Key	AT30TS75A-XM8M-TCT-ND	SENSOR TEMP I2C/SMBUS 8MSOP
U19	1		DFN - 10	NXP USA Inc.	MMA8652FCR1	Digi-Key	MMA8652FCR1CT-ND	ACCELEROMETER 2-8G I2C 10DFN
U20	1		TPS62097	Texas Instruments	TPS62097RWKT	Digi-Key	296-43330-1-ND	IC REG BUCK ADJ 2A 11VQFN
USB-1, USB-2, USB-3	3		USB-MicroB	Amphenol FCI	10103594-0001LF	Digi-Key	609-4050-1-ND	CONN RCPT STD MICRO USB TYPE B
XTAL-1	1	12MHz	7M-12.000MEEQ-T	TXC CORPORATION	7M-12.000MEEQ-T	Digi-Key	887-1316-1-ND	CRYSTAL 12.0000MHZ 10PF SMD
XTAL-2	1	8 MHz	XTAL2PIN-5mmx3.2mm	TXC CORPORATION	7A-8.000MAAE-T	Digi-Key	887-1740-1-ND	CRYSTAL 8.0000MHZ 12PF SMD
XTAL-3	1	32.768 kHz	XTAL2PIN-5mmx3.2mm	Abracon LLC	ABS10-32.768KHZ-1-T	Digi-Key	535-9806-1-ND	CRYSTAL 32.7680KHZ 12.5PF SMD
XTAL-4	1		Crsytal 11.5 x 3.5mm					
	416							

**Approved**	**Notes**
