# Supplementary material for: MIGOU: A Low-Power Experimental Platform with Programmable Logic Resources and Software-Defined Radio Capabilities
Source: Sensors (Basel). 2019 Nov 15;19(22):4983. doi: 10.3390/s19224983 (PMC6891619; doi:10.3390/s19224983)
Supplement: Supplementary file 1 [file sensors-19-04983-s001.zip › MIGOU-Schematics.pdf]

MIGOU PLATFORM

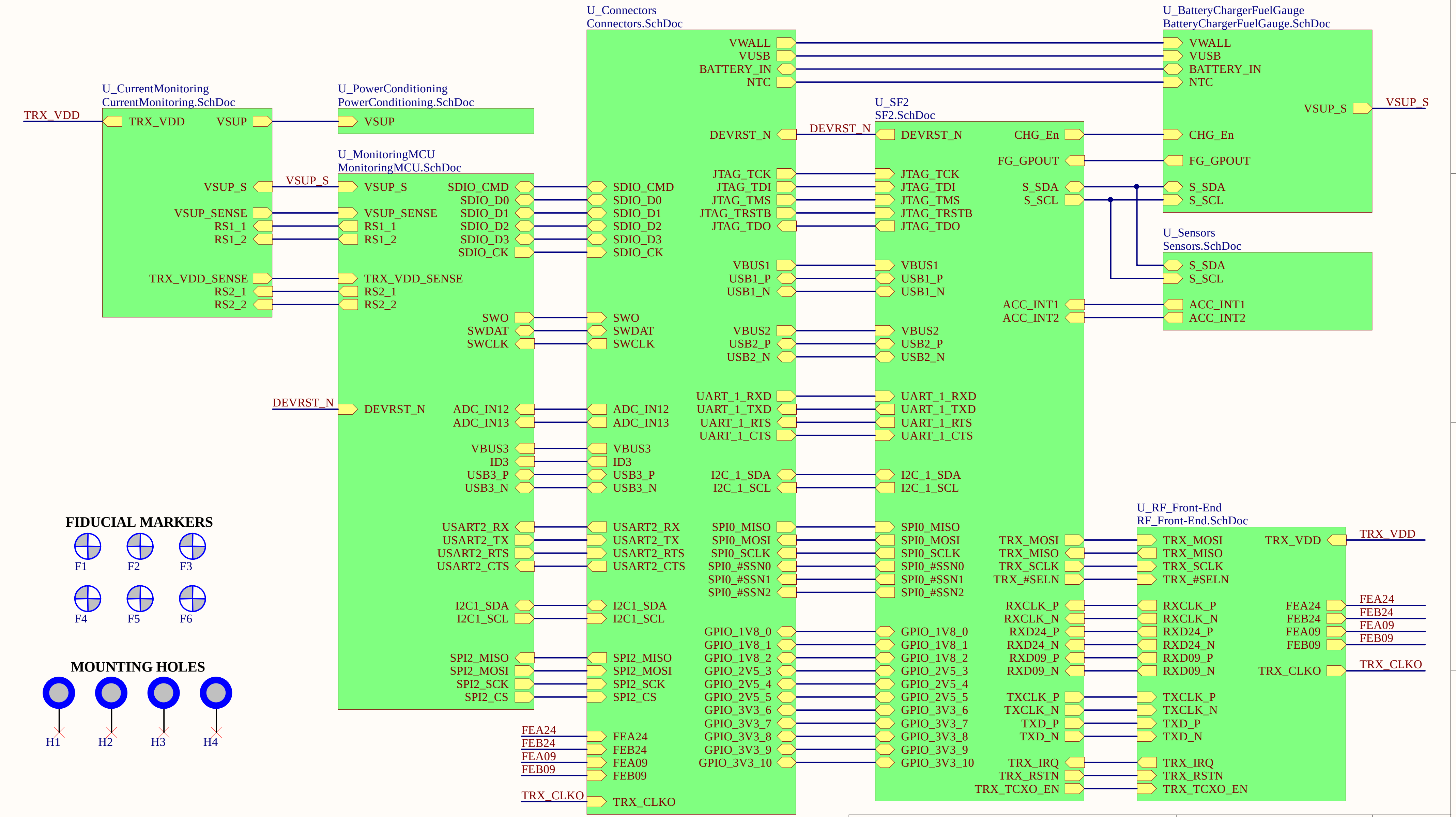

© 2019 by the authors. This work is licensed under the Creative Commons Attribution 4.0 (CC BY) International License (http://creativecommons.org/licenses/by/4.0/).

Title: *MIGOU PLATFORM*		***B105 Electronic Systems Lab*** *ETSI Telecomunicación, B-105* *Universidad Politécnica de Madrid* *Avda. Complutense, 30* *28040 Madrid - Spain*
Engineers: *Ramiro Utrilla and José Martín*	Revision: *v1r0*	
Date: *22/10/2019* Time: *11:31:29*	Sheet: *1* of *14*	
File: *D:\Repositorio\migou-platform\MIGOU\Schematics\MIGOUplatform.SchDoc*		

CONNECTORS

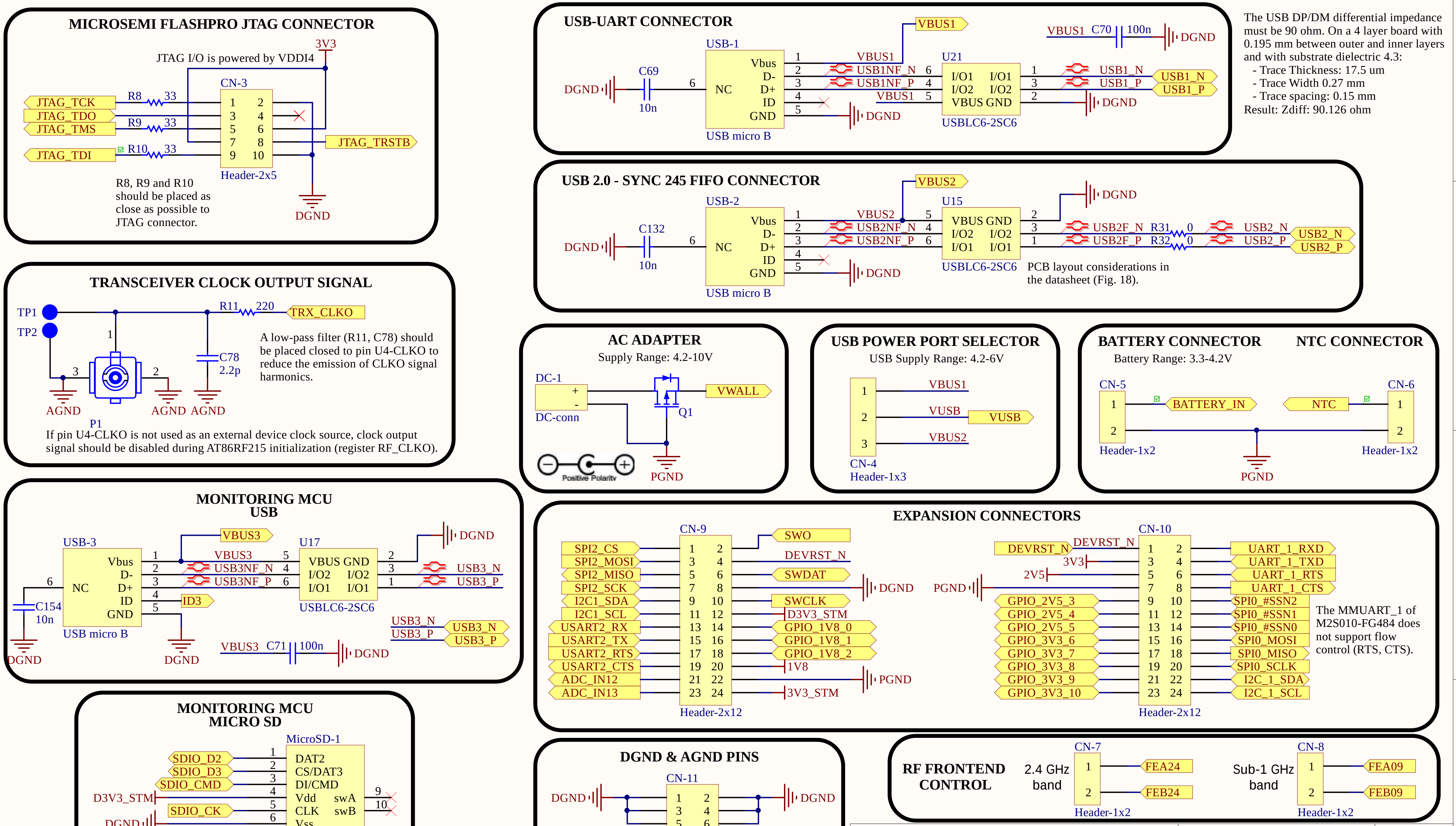

Title: *CONNECTORS*

Engineers: *Ramiro Utrilla and José Martín* | Revision: *v1r0*

Date: *22/10/2019* | Time: *11:31:30* | Sheet: *2* of *14*

File: *D:\Repositorio\migou-platform\MIGOU\Schematics\Connectors.SchDoc*

B105 Electronic Systems Lab
ETSI Telecomunicación, B-105
Universidad Politécnica de Madrid
Avda. Complutense, 30
28040 Madrid - Spain

BATTERY AND POWER PATH MAN.

BATTERY FUEL GAUGE

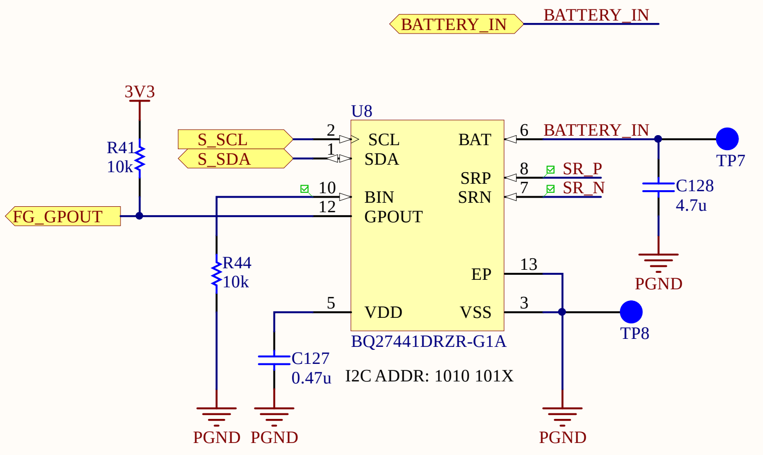

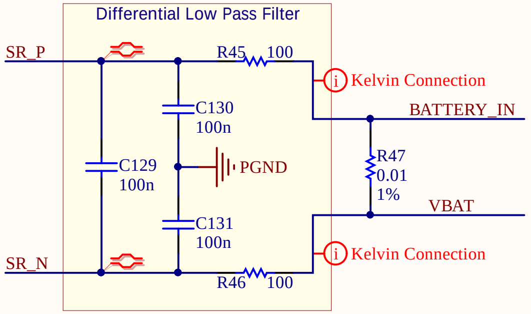

Detailed information about the Differential Low Pass Filter in the following application report:
Single Cell Gas Gauge Circuit Design (SLUA456 - March 2008)

Detailed information about PCB layout in the following application report:
Single Cell Impedance Track Printed-Circuit Board Layout Guide (SLUA457 - March 2007)

BATTERY CHARGER

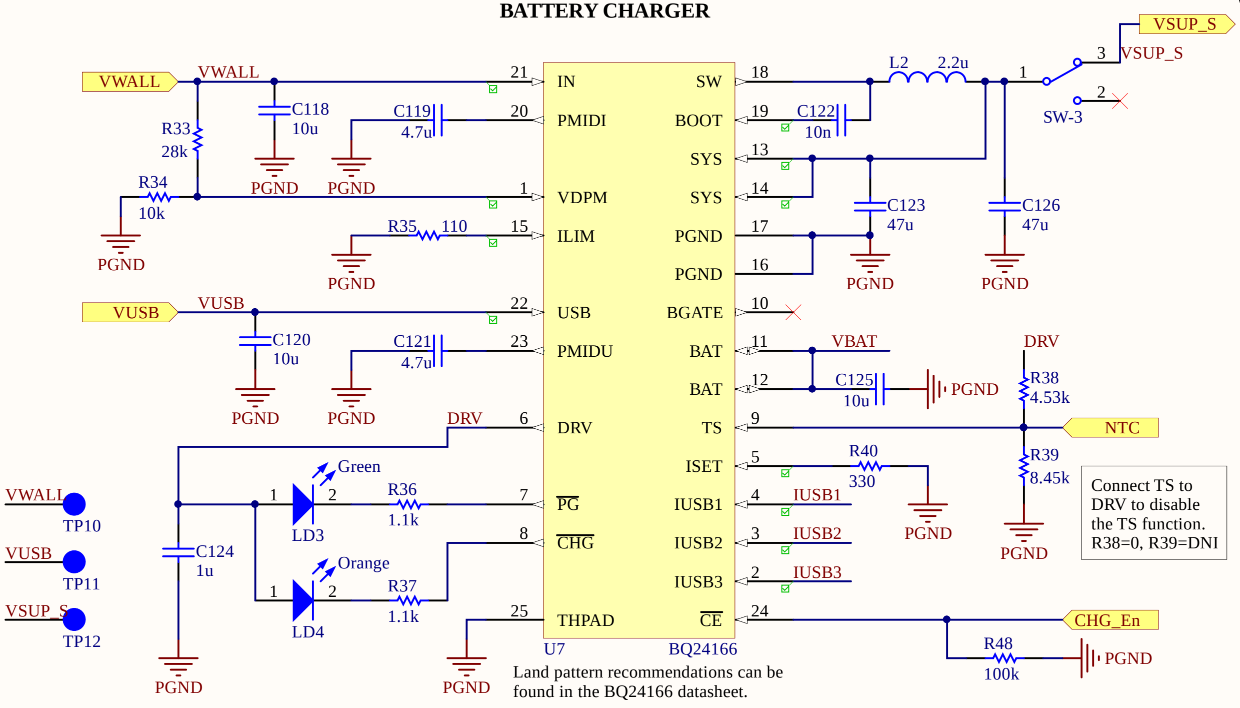

Connect TS to DRV to disable the TS function. R38=0, R39=DNI

Land pattern recommendations can be found in the BQ24166 datasheet.

CHARACTERISTICS:
- Overvoltage and overcurrent protection.
- Battery short circuit protection.
- IN port:
  - Maximum current: 2.5 A
  - Operating voltage range: 4.2 - 10 V
- USB port:
  - Maximum current: 1 A
  - Operating voltage range: 4.2 - 6 V
- BAT port:
  - Max. chg/dischg current: 2.5 A

CONFIGURATION:
- Vin_dpm= 4.56V with R33 = 28 kohm
- I_INlim = 2.4 A with R35 = 110 ohm
- I_USBlim = 0.5 A with IUSB1 = 1, IUSB2 = 0, IUSB3 = 0
- I_charge = 1.5A with R40 = 330 ohm
- NTC: NXFT15XV103FA2B150 => R38 = 4,53k and R39 = 8,45k

The BQ24166 is a switch mode single cell Li-Ion/Li-Polymer battery charger with power path management. It has dual inputs which allow operation from either a USB port or an AC adapter.

SYS output powers the external system load connected to the battery. This output is active whenever a source is connected to IN, USB or BAT.

USB Input Current Limit

IUSB3	IUSB2	IUSB1	Current Limit
0	0	0	100 mA
0	0	1	500 mA
0	1	0	1.5 A
0	1	1	Hi-Z Mode
1	0	0	150 mA
1	0	1	900 mA
1	1	0	800 mA
1	1	1	Hi-Z Mode

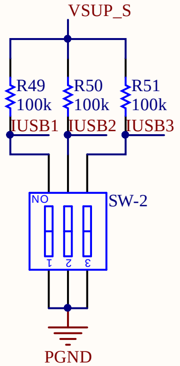

POWER LED

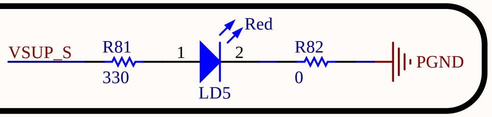

Title:	*BATTERY AND POWER PATH MAN.*
Engineers:	*Ramiro Utrilla and José Martín*
Revision:	*v1r0*
Date:	*22/10/2019*
Time:	*11:31:31*
Sheet:	*3* of *14*
File:	*D:\Repositorio\migou-platform\MIGOU\Schematics\BatteryChargerFuelGauge.SchDoc*

B105 Electronic Systems Lab
ETSI Telecomunicación, B-105
Universidad Politécnica de Madrid
Avda. Complutense, 30
28040 Madrid - Spain

CURRENT MONITORING

VSUP CURRENT MONITORING

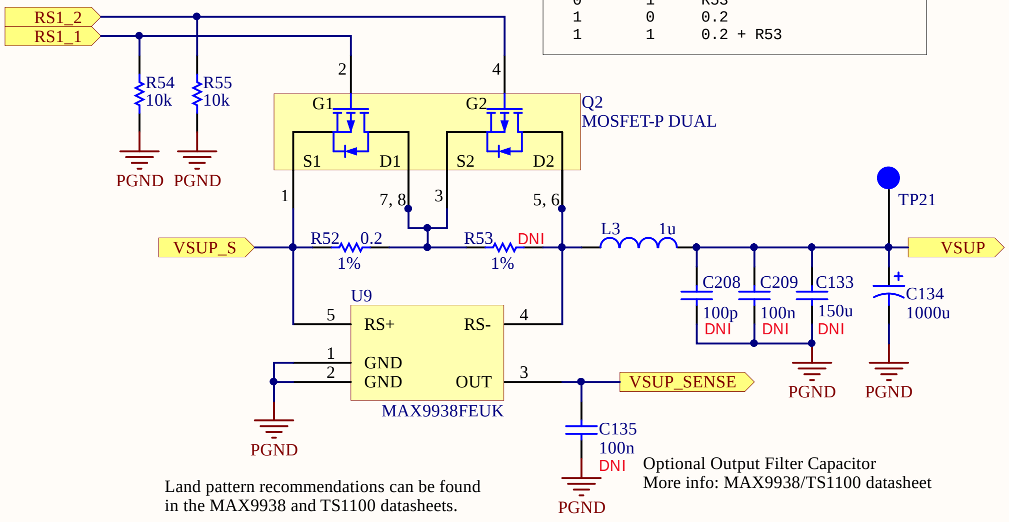

Rsense Combinations

RS1_1	RS1_2	Rsense
0	0	0.014 -> MOSFETs R_DS_ON
0	1	R53
1	0	0.2
1	1	0.2 + R53

Land pattern recommendations can be found in the MAX9938 and TS1100 datasheets.

Optional Output Filter Capacitor
More info: MAX9938/TS1100 datasheet

SPECIFICATIONS:

VSUP_S Range:	3.5 - 4.2 V
Current Sensing Range:	300 uA - 300 mA
MAX9938 Gain:	50 V/V
Vsense for the full-scale:	60 mV (for gain of 50 V/V)
R_DS_ON @ V_GS = 3.3V	0.0070 Ohm

DESIGN:

Rsense (Vsense_fs/Iload_max):	0.2 Ohm
P_max = 60 mV x 300 mA =	18 mW

When:
Iload_max=300 mA => Vsense = 60 mV => Vout_max = 3 V
Iload_min=300 uA => Vsense = 60 uV => Vout_min = 3 mV

ADC CHARACTERISTICS:

Resolution:	12-bit
Input Range:	0 - 3.6 V
Min. Voltage Step:	1 mV
Min. Current Step:	100uA

TRX_VDD CURRENT MONITORING

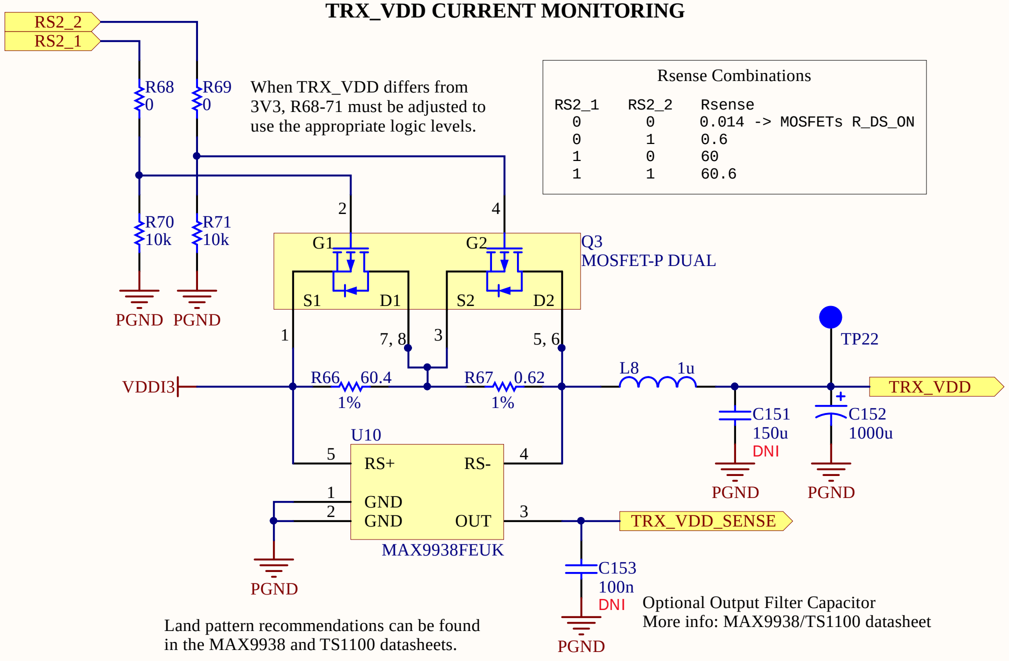

When TRX_VDD differs from 3V3, R68-71 must be adjusted to use the appropriate logic levels.

Rsense Combinations

RS2_1	RS2_2	Rsense
0	0	0.014 -> MOSFETs R_DS_ON
0	1	0.6
1	0	60
1	1	60.6

Land pattern recommendations can be found in the MAX9938 and TS1100 datasheets.

Optional Output Filter Capacitor
More info: MAX9938/TS1100 datasheet

SPECIFICATIONS:

TRX_VDD possible values:	1.8 / 2.5 / 3.3 V
Current Sensing Range:	1 uA - 100 mA
- First half (with R66):	1 uA - 1 mA
- Second half (with R67):	100 uA - 100 mA
MAX9938 Gain:	50 V/V
Vsense_max:	60 mV (for gain of 50 V/V)
R_DS_ON @ V_GS = 4.5V	0.0070 Ohm

DESIGN:

Rsense (Vsense_fs/Iload_max):	
- First half:	60 Ohm
- Second half:	0.6 Ohm
P_max = 60 mV x 100 mA =	6 mW

Using Rsense=60 Ohm, when:
Iload_max=1 mA => Vsense = 60 mV => Vout_max = 3 V
Iload_min=1 uA => Vsense = 60 uV => Vout_min = 3 mV

Using Rsense=0.6 Ohm, when:
Iload_max=100 mA => Vsense = 60 mV => Vout_max = 3 V
Iload_min=100 uA => Vsense = 60 uV => Vout_min = 3 mV

ADC CHARACTERISTICS:

Resolution:	12-bit
Input Range:	0 - 3.6 V
Min. Voltage Step:	1 mV
Min. Current Step:	
- With Rsense=60 Ohm:	333nA
- With Rsense=0.6 Ohm:	33uA

Title: *CURRENT MONITORING*
Engineers: *Ramiro Utrilla and José Martín* Revision: *v1r0*
Date: *22/10/2019* Time: *11:31:33* Sheet: *4* of *14*
File: *D:\Repositorio\migou-platform\MIGOU\Schematics\CurrentMonitoring.SchDoc*

B105 Electronic Systems Lab
ETSI Telecomunicación, B-105
Universidad Politécnica de Madrid
Avda. Complutense, 30
28040 Madrid - Spain

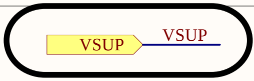

POWER CONDITIONING

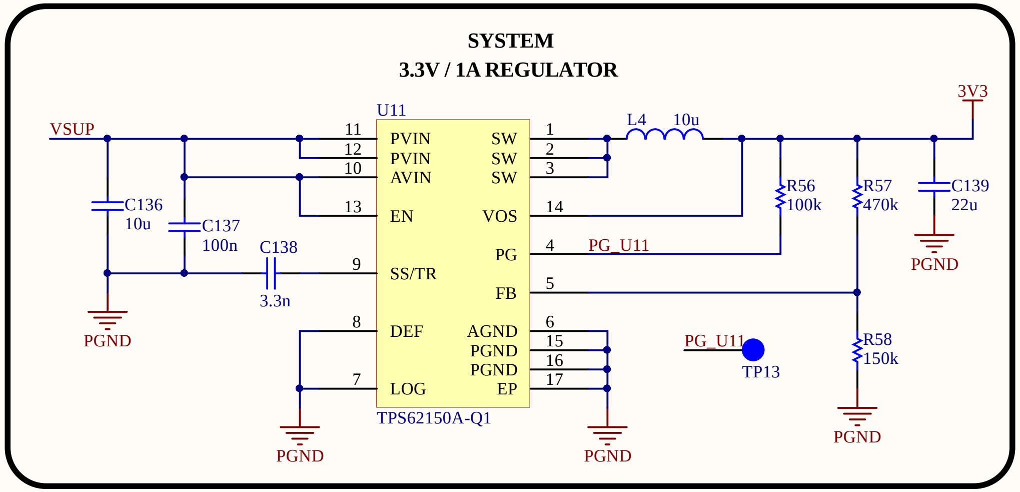

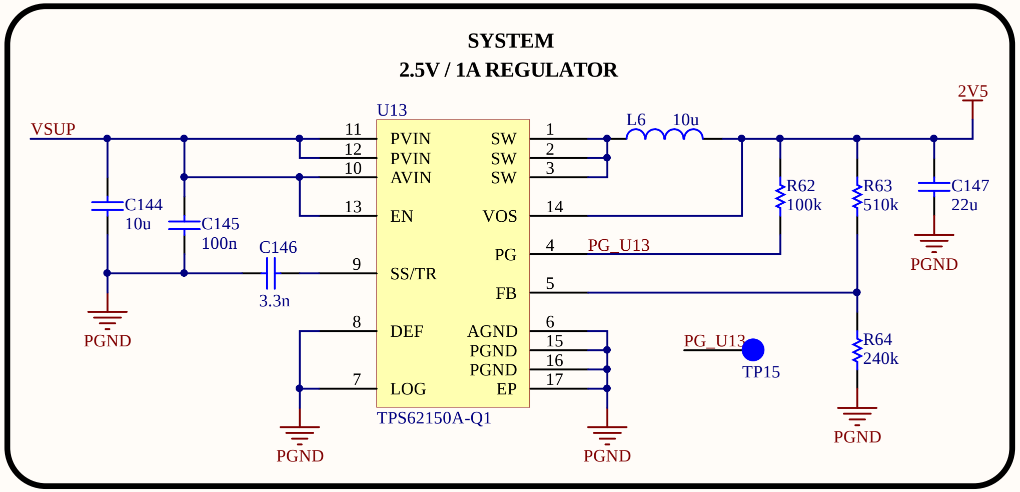

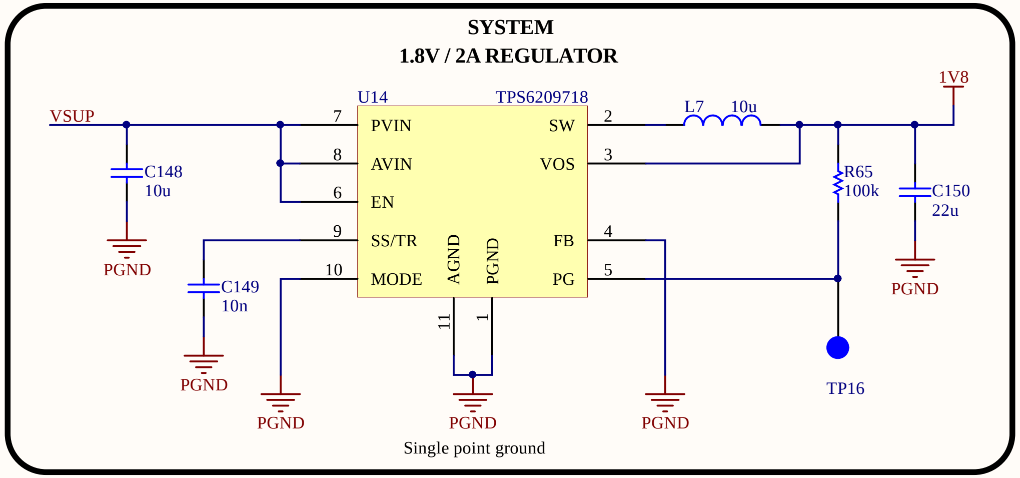

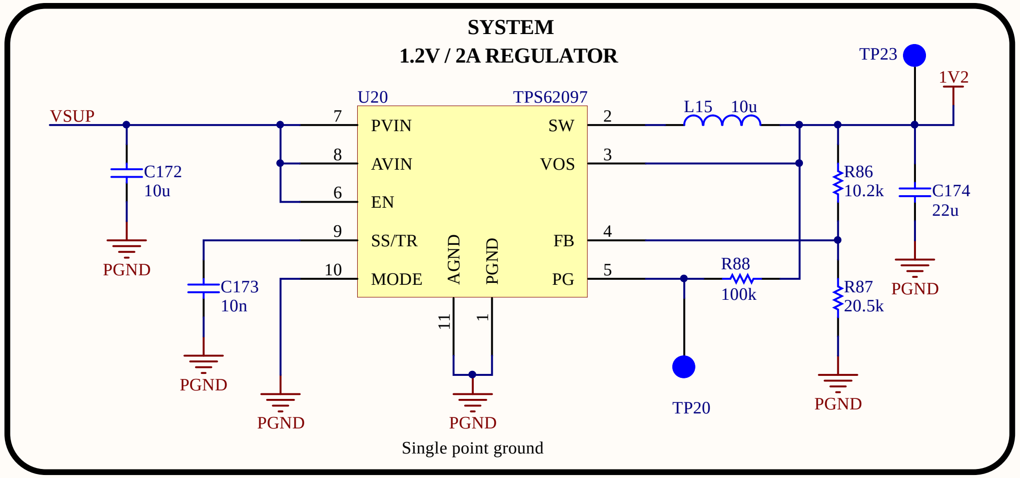

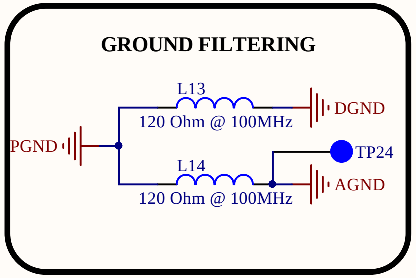

TPS62150A-Q1 DESIGNS:

Vin range:	3 - 17 V
Vout:	3.3 V / 2.5 V
Iout max.:	1 A

More info (Layout guidelines, filtering...):
- TPS62150A-Q1 Datasheet
- TI - AN - SLVA463A
- TI - AN - SLYT614
- TI - UG - SLVU437A

TPS62097 DESIGNS:

Vin range:	2.5 - 6 V
Vout:	1.8 V / 1.2 V
Iout max.:	2 A
Startup time:	1 ms with C149 and C173 = 10 nF

More info (Layout guidelines, filtering...):
- TPS62097 Datasheet (FB pin description)
- TI - AN - SLVA710

NOTE:
L4, L5 (MonitoringMCU.SchDoc), L6, L7 and L15 have the same layout, and it is compatible with the following inductors:
- 74438356022 (WE)
- XFL4020-222MEB
- IHLP1212BZER2R2M11

Title: *POWER CONDITIONING*

Engineers: *Ramiro Utrilla and José Martín* Revision: *v1r0*

Date: *22/10/2019* Time: *11:31:34* Sheet: *5* of *14*

File: *D:\Repositorio\migou-platform\MIGOU\Schematics\PowerConditioning.SchDoc*

B105 Electronic Systems Lab
ETSI Telecomunicación, B-105
Universidad Politécnica de Madrid
Avda. Complutense, 30
28040 Madrid - Spain

MONITORING MCU

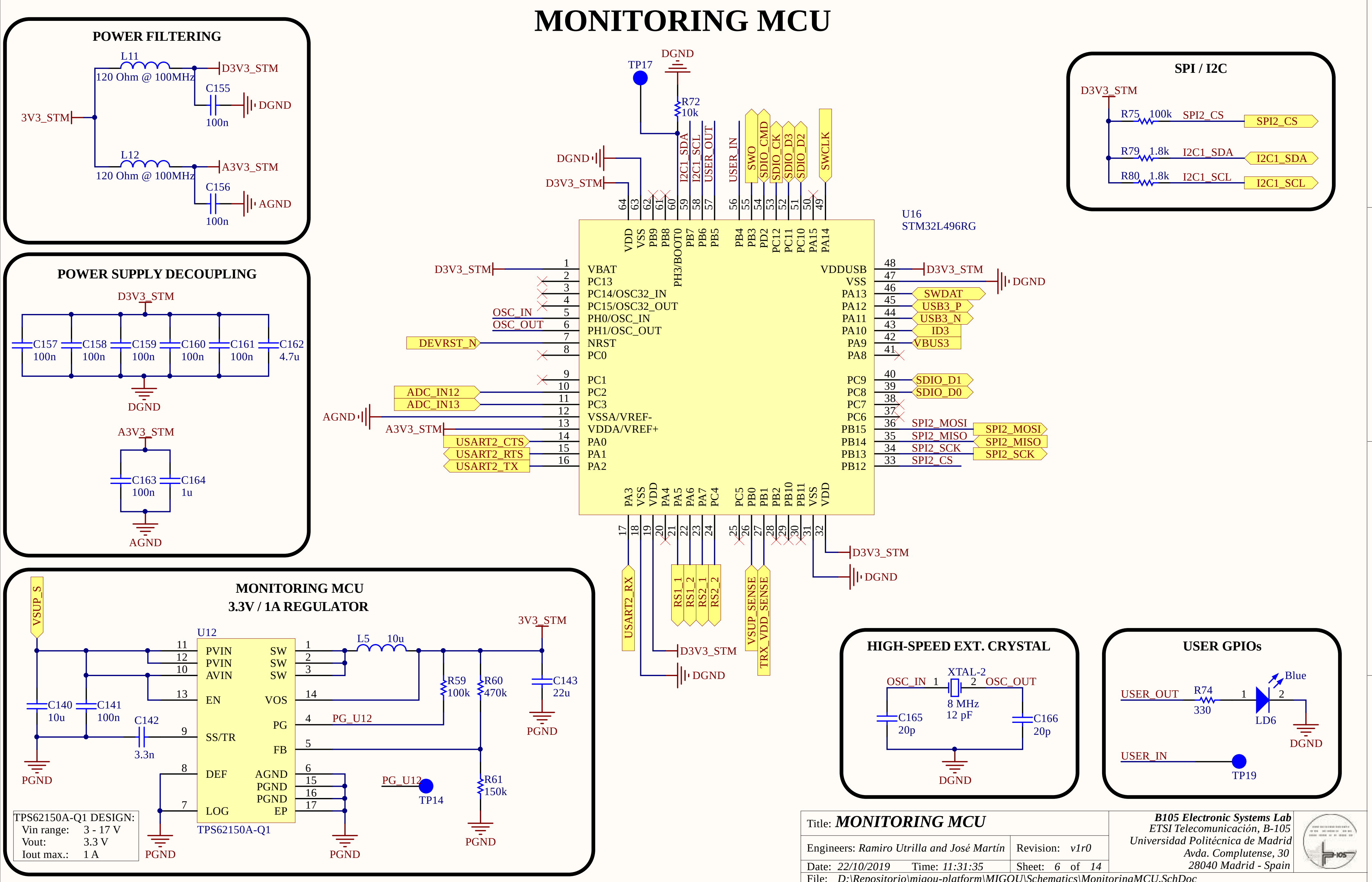

Title: *MONITORING MCU*	*B105 Electronic Systems Lab*
Engineers: *Ramiro Utrilla and José Martín* — Revision: *v1r0*	*ETSI Telecomunicación, B-105* *Universidad Politécnica de Madrid* *Avda. Complutense, 30* *28040 Madrid - Spain*
Date: *22/10/2019* — Time: *11:31:35* — Sheet: *6* of *14*	
File: *D:\Repositorio\migou-platform\MIGOU\Schematics\MonitoringMCU.SchDoc*	

SmartFusion2

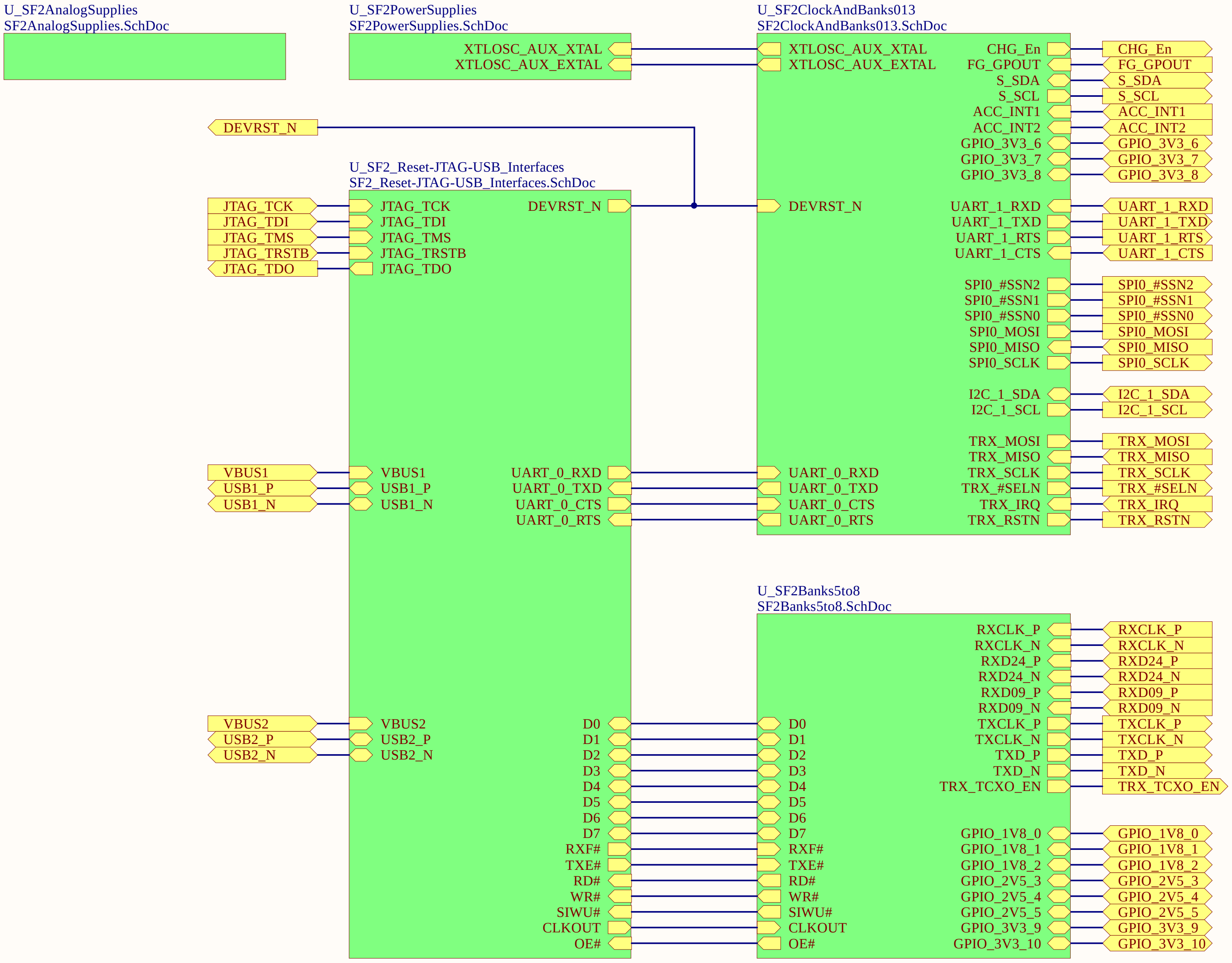

Title: ***SmartFusion2***

Engineers: *Ramiro Utrilla and José Martín* Revision: *v1r0*

Date: *22/10/2019* Time: *11:31:36* Sheet: *7* of *14*

File: *D:\Repositorio\migou-platform\MIGOU\Schematics\SF2.SchDoc*

B105 Electronic Systems Lab
ETSI Telecomunicación, B-105
Universidad Politécnica de Madrid
Avda. Complutense, 30
28040 Madrid - Spain

SF2 POWER SUPPLIES

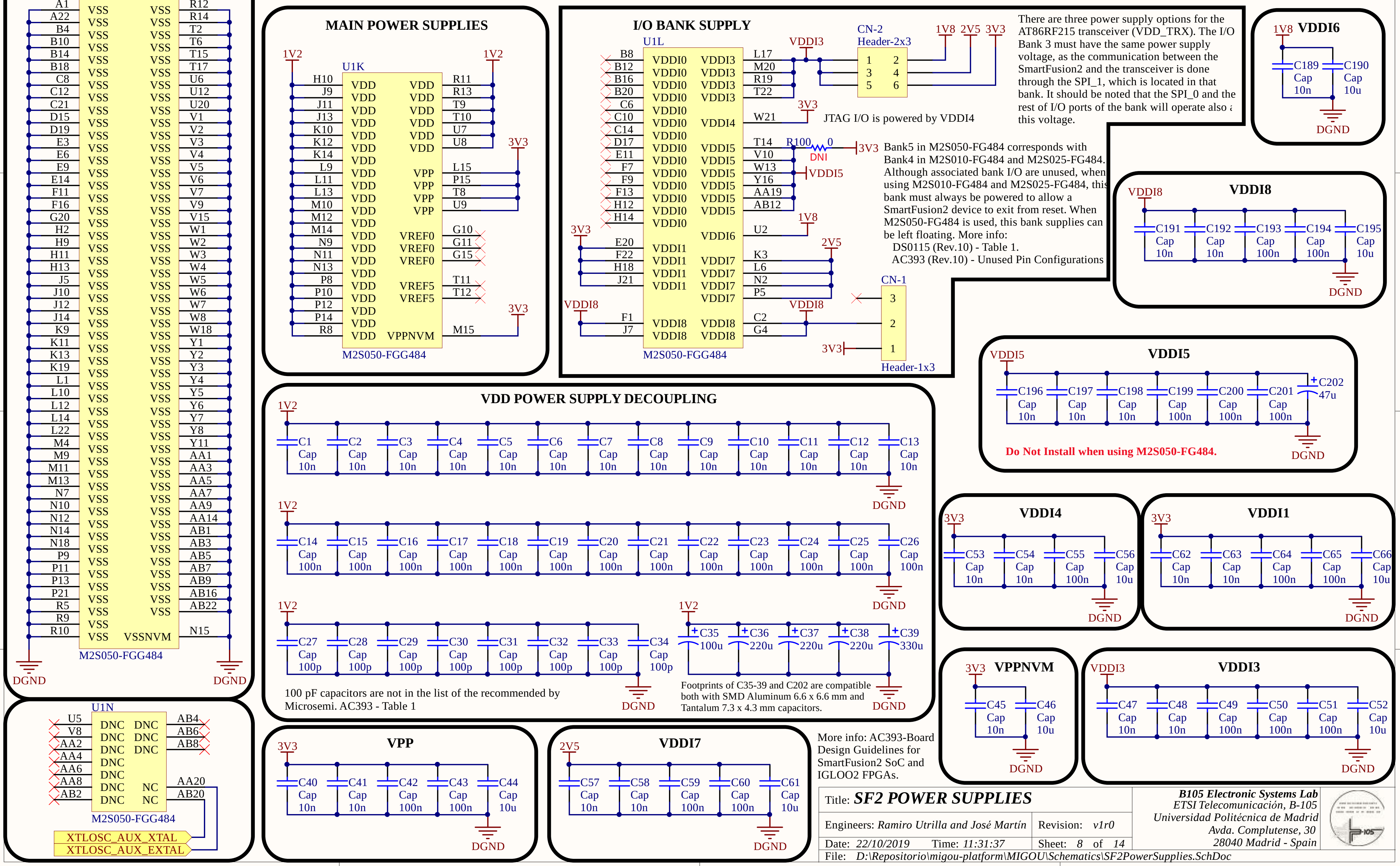

Title: ***SF2 POWER SUPPLIES***		***B105 Electronic Systems Lab*** *ETSI Telecomunicación, B-105* *Universidad Politécnica de Madrid* *Avda. Complutense, 30* *28040 Madrid - Spain*
Engineers: *Ramiro Utrilla and José Martín*	Revision: *v1r0*	
Date: *22/10/2019* Time: *11:31:37*	Sheet: *8* of *14*	
File: *D:\Repositorio\migou-platform\MIGOU\Schematics\SF2PowerSupplies.SchDoc*		

SF2 ANALOG SUPPLIES

If the associated PLL is used as a clock multiplier, these supplies must be connected over the RC filter circuitry between the common PLL supply and the corresponding on-board PLL return path. If the PLL is unused or used as a clock divider, these supplies can be connected directly to either 2.5V or 3.3V without filter circuitry.

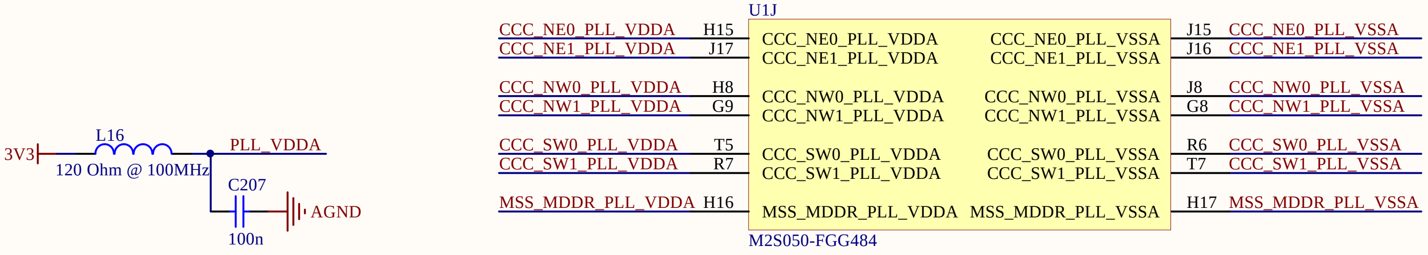

VSSA pins provide the internal PLL current return path for VDDAs. These pins must be connected to the corresponding PLL_VDDA through an RC filter circuit. PLL_VSSA and VSS are shorted internally in the silicon. Therefore, to filter noise and control long-term jitter, PLL_VSSA must NOT be connected to an external ground.

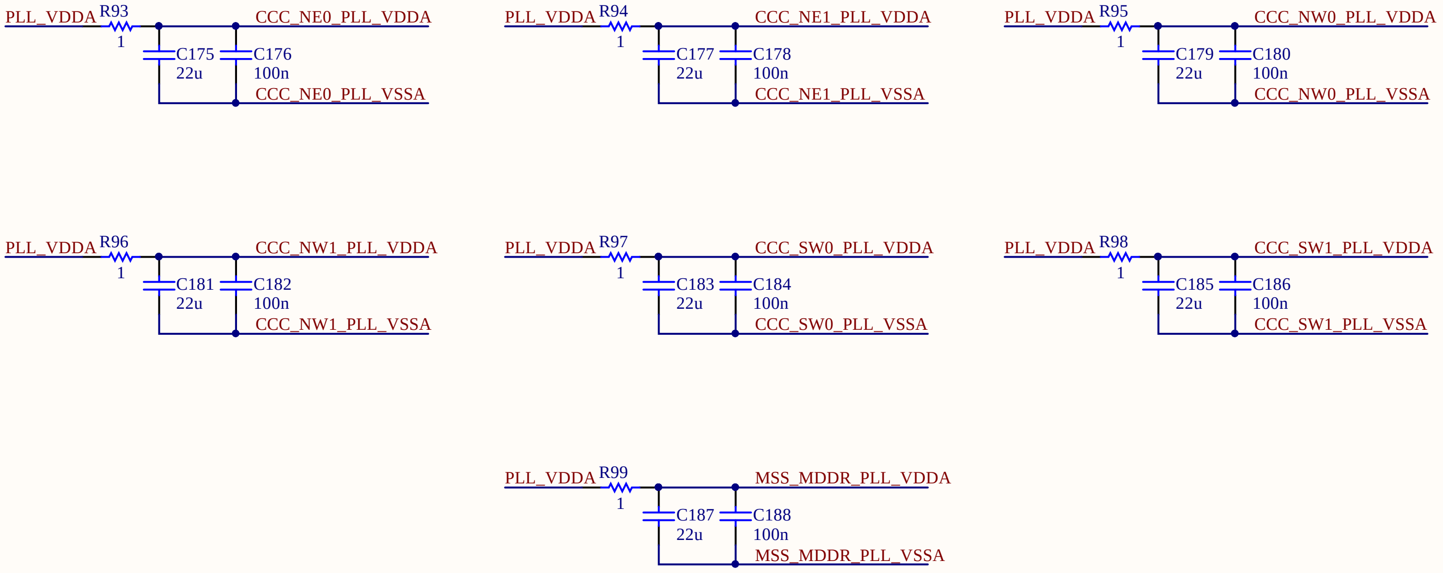

Title: *SF2 ANALOG SUPPLIES*

Engineers: *Ramiro Utrilla and José Martín* Revision: *v1r0*

Date: *22/10/2019* Time: *11:31:39* Sheet: *9* of *14*

File: *D:\Repositorio\migou-platform\MIGOU\Schematics\SF2AnalogSupplies.SchDoc*

B105 Electronic Systems Lab
ETSI Telecomunicación, B-105
Universidad Politécnica de Madrid
Avda. Complutense, 30
28040 Madrid - Spain

SF2 CLOCK AND BANKS 0, 1 AND 3

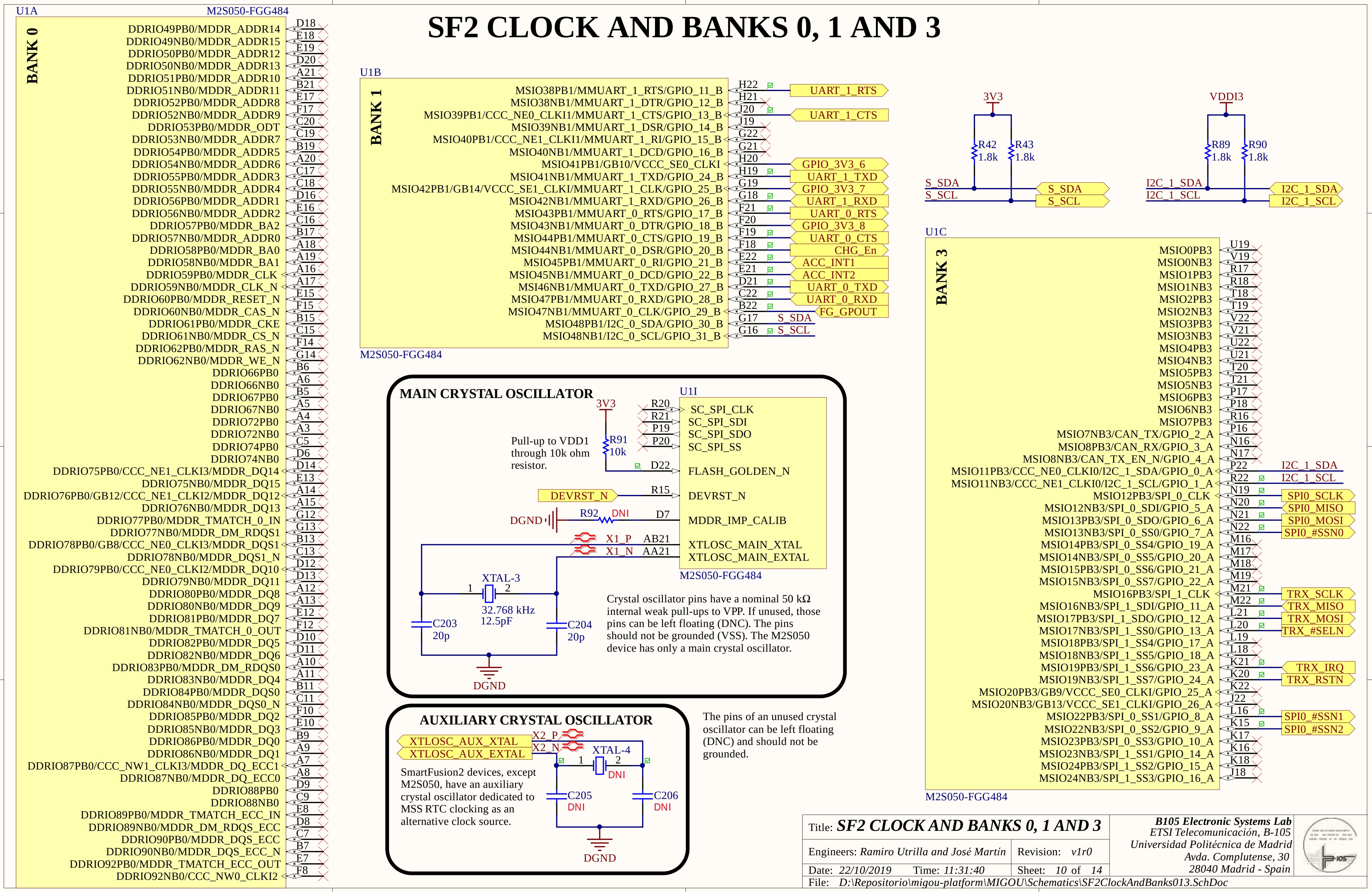

SF2 BANKS 5 TO 8

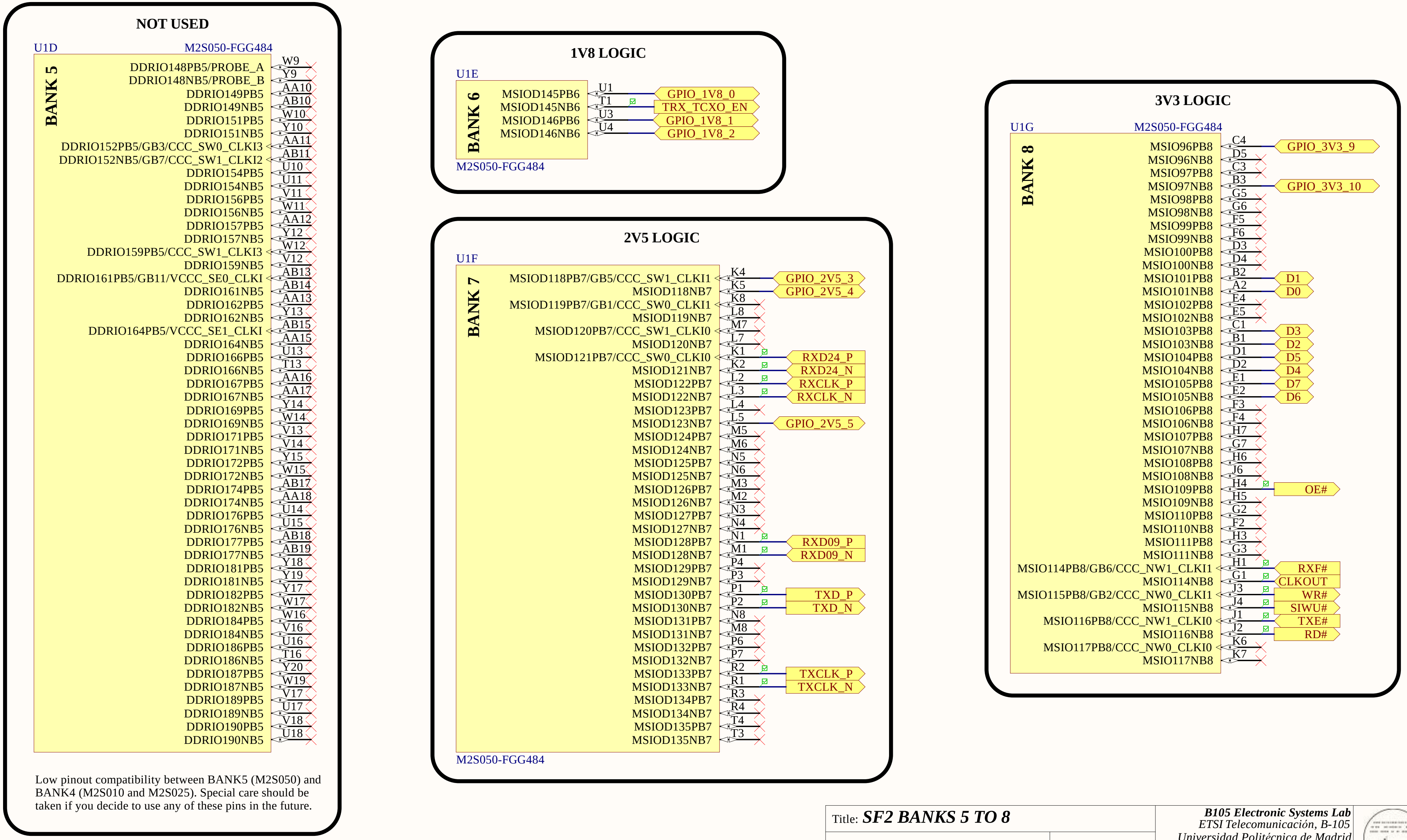

Title: ***SF2 BANKS 5 TO 8***	
Engineers: *Ramiro Utrilla and José Martín*	Revision: *v1r0*
Date: *22/10/2019* Time: *11:31:41*	Sheet: *11* of *14*
File: *D:\Repositorio\migou-platform\MIGOU\Schematics\SF2Banks5to8.SchDoc*	

B105 Electronic Systems Lab
ETSI Telecomunicación, B-105
Universidad Politécnica de Madrid
Avda. Complutense, 30
28040 Madrid - Spain

SF2 RESET, JTAG AND USBs

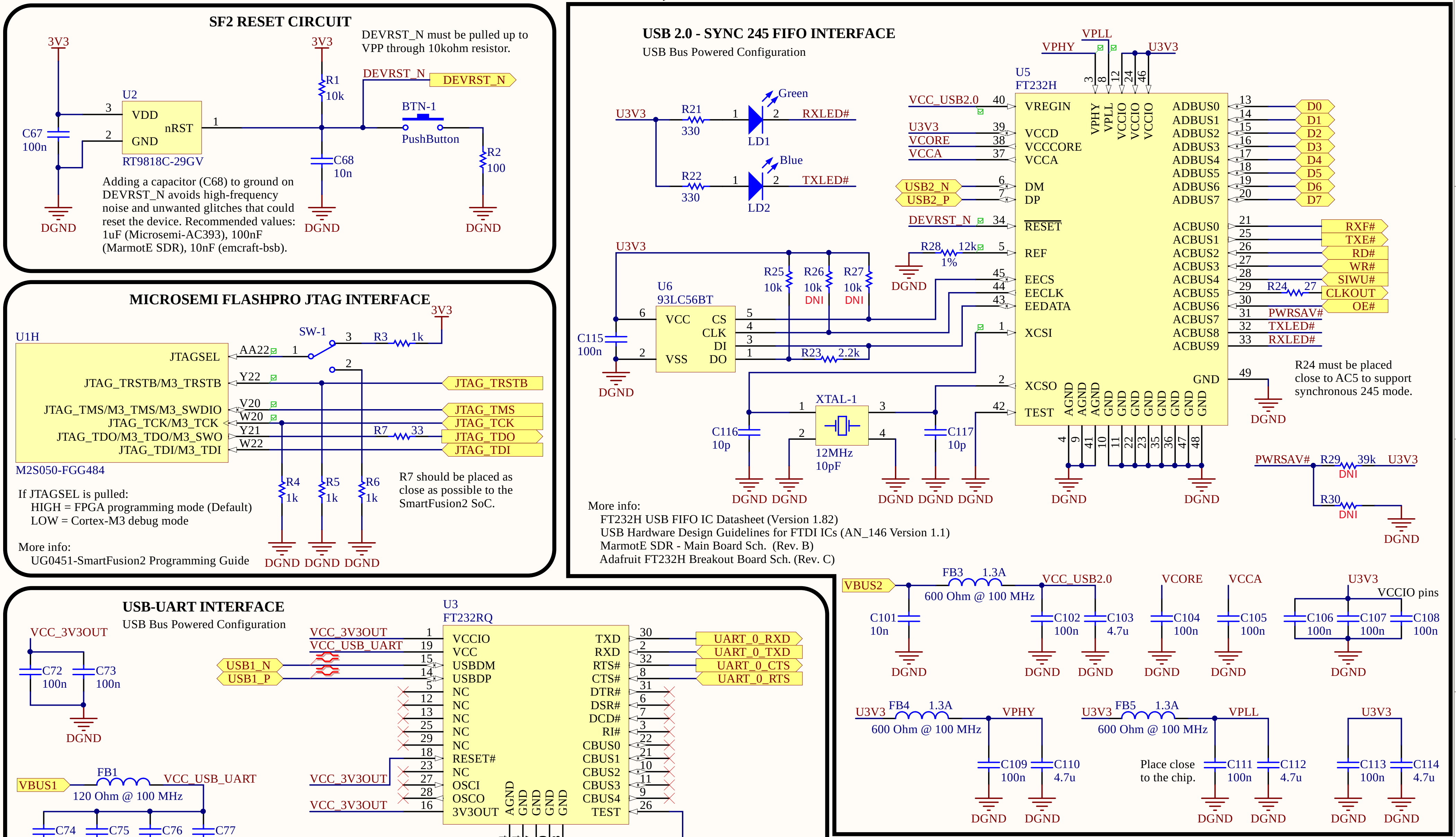

Title: *SF2 RESET, JTAG AND USBs*

Engineers: *Ramiro Utrilla and José Martín* — Revision: *v1r0*

Date: *22/10/2019* — Time: *11:31:43* — Sheet: *12* of *14*

File: *D:\Repositorio\migou-platform\MIGOU\Schematics\SF2_Reset-JTAG-USB_Interfaces.SchDoc*

B105 Electronic Systems Lab
ETSI Telecomunicación, B-105
Universidad Politécnica de Madrid
Avda. Complutense, 30
28040 Madrid - Spain

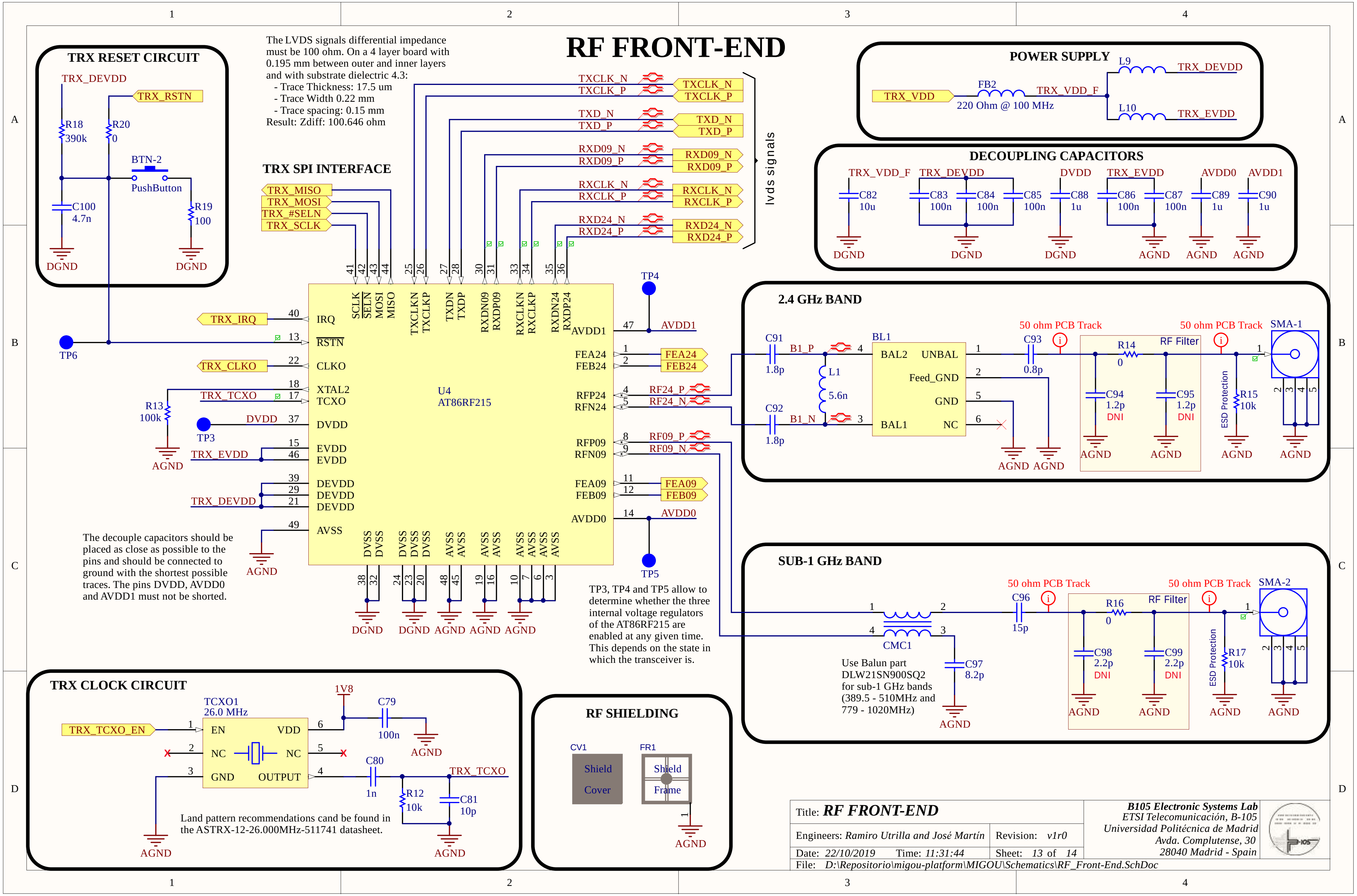
RF FRONT-END
TRX RESET CIRCUIT
TRX_DEVDD
TRX_RSTN
R18 390k
R20 0
BTN-2 PushButton
C100 4.7n
R19 100
DGND
The LVDS signals differential impedance must be 100 ohm. On a 4 layer board with 0.195 mm between outer and inner layers and with substrate dielectric 4.3:
- Trace Thickness: 17.5 um
- Trace Width 0.22 mm
- Trace spacing: 0.15 mm
Result: Zdiff: 100.646 ohm
TRX SPI INTERFACE
TRX_MISO
TRX_MOSI
TRX_#SELN
TRX_SCLK
TXCLK_N
TXCLK_P
TXD_N
TXD_P
RXD09_N
RXD09_P
RXCLK_N
RXCLK_P
RXD24_N
RXD24_P
lvds signals
TRX_IRQ
TP6
TRX_CLKO
TRX_TCXO
R13 100k
TP3
DVDD
TRX_EVDD
TRX_DEVDD
AGND
U4 AT86RF215
IRQ
RSTN
CLKO
XTAL2
TCXO
DVDD
EVDD
DEVDD
AVSS
SCLK SELN MOSI MISO TXCLKN TXCLKP TXDN TXDP RXDN09 RXDP09 RXCLKN RXCLKP RXDN24 RXDP24
AVDD1
FEA24
FEB24
RFP24
RFN24
RFP09
RFN09
FEA09
FEB09
AVDD0
DVSS DVSS DVSS DVSS DVSS AVSS AVSS AVSS AVSS AVSS AVSS AVSS AVSS
TP4
AVDD1
RF24_P
RF24_N
RF09_P
RF09_N
AVDD0
TP5
The decouple capacitors should be placed as close as possible to the pins and should be connected to ground with the shortest possible traces. The pins DVDD, AVDD0 and AVDD1 must not be shorted.
TP3, TP4 and TP5 allow to determine which of the three internal voltage regulators of the AT86RF215 are enabled at any given time. This depends on the state in which the transceiver is.
POWER SUPPLY
TRX_VDD
FB2 220 Ohm @ 100 MHz
TRX_VDD_F
L9
TRX_DEVDD
L10
TRX_EVDD
DECOUPLING CAPACITORS
TRX_VDD_F TRX_DEVDD DVDD TRX_EVDD AVDD0 AVDD1
C82 10u
C83 100n
C84 100n
C85 100n
C88 1u
C86 100n
C87 100n
C89 1u
C90 1u
DGND DGND DGND AGND AGND AGND
2.4 GHz BAND
C91 1.8p
B1_P
C92 1.8p
B1_N
L1 5.6n
BL1
BAL2 UNBAL
Feed_GND
GND
BAL1 NC
C93 0.8p
50 ohm PCB Track
RF Filter
R14 0
C94 1.2p DNI
C95 1.2p DNI
ESD Protection
R15 10k
SMA-1
SUB-1 GHz BAND
CMC1
C97 8.2p
C96 15p
R16 0
C98 2.2p DNI
C99 2.2p DNI
R17 10k
SMA-2
Use Balun part DLW21SN900SQ2 for sub-1 GHz bands (389.5 - 510MHz and 779 - 1020MHz)
TRX CLOCK CIRCUIT
TCXO1 26.0 MHz
TRX_TCXO_EN
EN VDD
NC NC
GND OUTPUT
1V8
C79 100n
C80 1n
R12 10k
C81 10p
TRX_TCXO
Land pattern recommendations cand be found in the ASTRX-12-26.000MHz-511741 datasheet.
RF SHIELDING
CV1 Shield Cover
FR1 Shield Frame
Title: RF FRONT-END
Engineers: Ramiro Utrilla and José Martín
Revision: v1r0
Date: 22/10/2019
Time: 11:31:44
Sheet: 13 of 14
File: D:\Repositorio\migou-platform\MIGOU\Schematics\RF_Front-End.SchDoc
B105 Electronic Systems Lab
ETSI Telecomunicación, B-105
Universidad Politécnica de Madrid
Avda. Complutense, 30
28040 Madrid - Spain

SENSORS

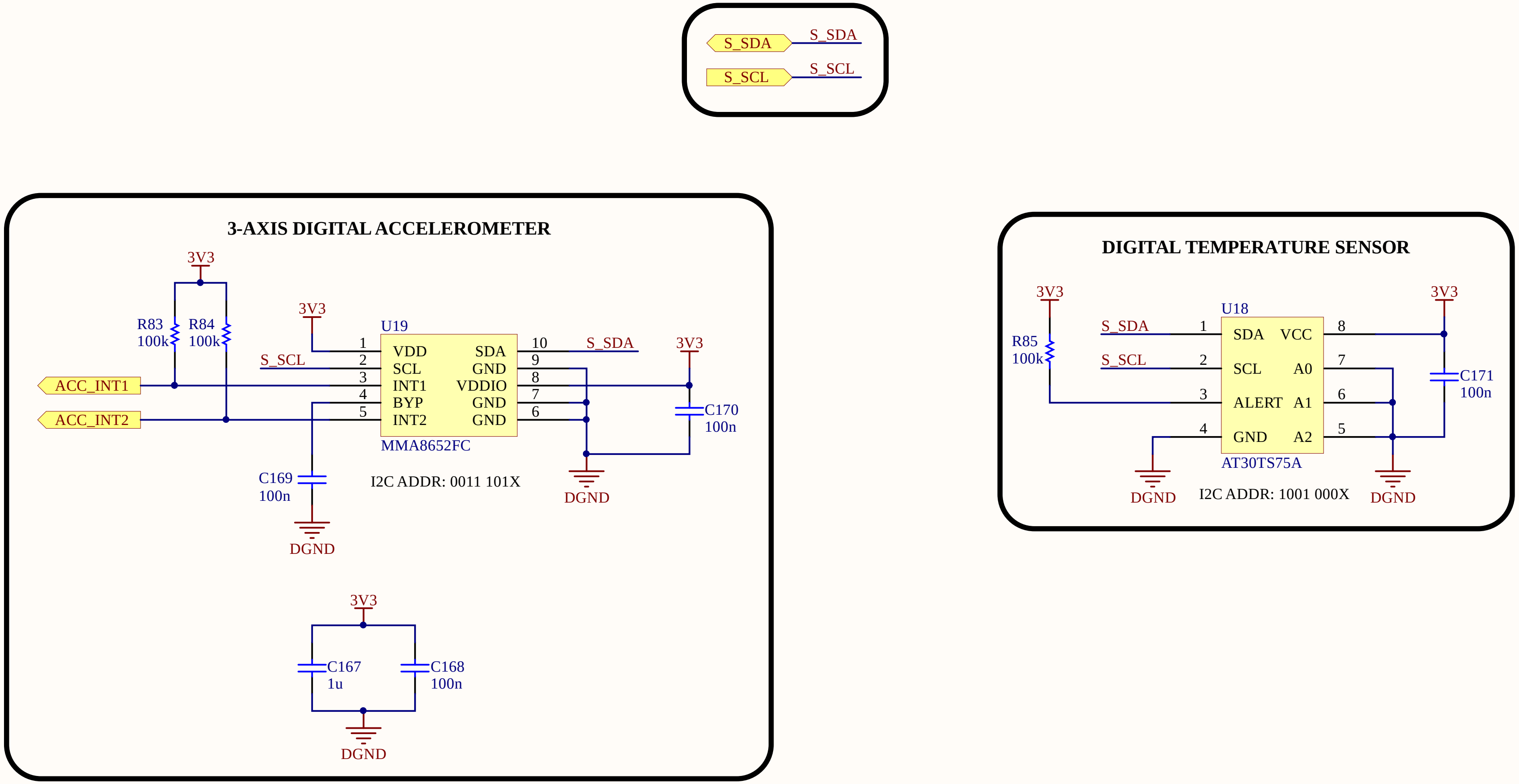

Title: ***SENSORS***			***B105 Electronic Systems Lab*** *ETSI Telecomunicación, B-105* *Universidad Politécnica de Madrid* *Avda. Complutense, 30* *28040 Madrid - Spain*
Engineers: *Ramiro Utrilla and José Martín*		Revision: *v1r0*	
Date: *22/10/2019*	Time: *11:31:45*	Sheet: *14* of *14*	
File: *D:\Repositorio\migou-platform\MIGOU\Schematics\Sensors.SchDoc*			
